# Size structure of the coral *Stylophora pistillata* across reef flat zones in the central Red Sea

**DOI:** 10.1038/s41598-022-17908-3

**Published:** 2022-08-17

**Authors:** Walter A. Rich, Susana Carvalho, Ronald Cadiz, Gloria Gil, Karla Gonzalez, Michael L. Berumen

**Affiliations:** Red Sea Research Center, King Abdullah University of Science and Technology, Thuwal, Saudi Arabia

**Keywords:** Population dynamics, Coral reefs

## Abstract

Demographic analyses offer insight into the state of a population. Here, we surveyed different reef flat zones (exposed, midreef and sheltered) of six reefs over a cross-shelf gradient to characterize the population structure of *Stylophora pistillata*, a coral species which dominates reef flats in the central Red Sea. Phototransects were conducted at each reef flat zone, and the density of *S. pistillata*, the planar area of each colony, and the occurrence of partial mortality were calculated using the program ImageJ. Each colony was also assigned a color morph (yellow, purple or mixed colors). Density and mean size were extremely variable, both among reef flat zones and reefs, but overall, both metrics were lower on the midshelf reefs. The yellow color morph accounted for nearly 90% of colonies surveyed and dominated most reef flats assessed, with the exception of one site where 81% of colonies were purple morphs. There were no spatial trends in the percentage of colonies suffering partial mortality, but overall there is a positive correlation with size class and proportion of colonies with partial mortality. Despite few trends emerging from assessing individual parameters, a PERMANOVA analysis revealed differences among reef flat zones in most of the reefs, highlighting the importance of multivariate analysis. The data presented here serve as a baseline for monitoring and may identify possible future demographic changes to this important coral species in a region increasingly affected by bleaching events.

## Introduction

Coral reef ecosystems are underpinned by hermatypic coral species, which construct the reef structure providing habitat for approximately one quarter of marine life^1^. At a fundamental level the growth and maintenance of the reef environment are dependent on the demographic rates of individual coral species (e.g., reproductive output, recruitment and settlement rates, colony growth rates, population density, and mortality)^2^. In recent decades, coral species have been severely affected by anthropogenic disturbances, compromising the stability of their populations and the resilience of coral reefs worldwide.

Long-term monitoring is ideal for gathering information on trends of population growth and decline, which can shed light on how species respond to changes in environmental conditions or disturbances. However, long-term data often are not available for many regions, especially remote coral reefs or historically understudied systems such as the Red Sea^3^. Historically, coral percent cover has been used as the standard monitoring variable in benthic reef communities, and is a useful metric that encapsulates very general information about the state of a coral reef. However, this traditional approach obscures details that can be useful in understanding population dynamics. This method ignores the actual abundance and sizes of individual corals, since “percent coral cover” only measures the total space occupied by a coral category. Assessing size structure (e.g., the abundance of individuals within different size classes in a population) is a better tool for understanding the population trajectories of a species because it captures demographic information not contained in other methods like percent cover^4–6^. Therefore, simple metrics such as size structure can offer a more in-depth “snapshot” of the state of a population. Often, size structure is used to infer the state of coral populations under anthropogenic stressors, such as pollution^7^, climate change^8–11^, and fishing pressure^12,13^. Therefore, in an era of intense degradation as a result of local and global disturbances, a more comprehensive evaluation of the status of coral reefs, potential causes of decline, and projected trajectories is essential to guide mitigation and conservation measures.

Size structure comprises two key metrics: abundance and the size of all individuals^7,14^. In corals, abundance is the product of several demographic processes, including asexual reproduction, settlement and mortality rates, while the size of individual coral colonies is dependent on growth rates and partial mortality^15–17^. The resulting size frequency distribution provides a way to compare demographic information among different species, populations or sites^14^. For example, a size-frequency distribution that is negatively skewed indicates an overabundance of large individuals in a population, while positive skew denotes a population dominated by small individuals. Previous studies have suggested that negatively skewed size-frequency distributions are potentially indicative of a degraded reef^14,18^. The implication is that there are few young individuals to replenish older individuals in a population, and may be due to a lack of (or unsuccessful) settlement/recruitment or high mortality rates for smaller size classes. While skewness alone cannot indicate the nature of degradation, it can be relevant for future investigations to understand the cause of demographic shifts.

Coral size structure varies naturally in time and space. Abiotic factors such as depth^19,20^, temperature^21^ and water quality^22^ can influence coral size structure. Environmental conditions can vary drastically at the scale of the reef, particularly as a function of wave exposure and water depth; sites near the exposed side of a reef usually experience less variability than sheltered sites in a variety of parameters (e.g., temperature, oxygen concentration, etc.)^23^. Perhaps the most extreme gradient that exists over this small scale is over the shallow reef flat from exposed to sheltered sites. The reef flat is typically the shallowest part of the reef and its height is limited by sea level^24^. The reef flat habitat is subjected to frequent disturbances like low tides, high temperature variability, and extreme wave action^25^. Though the benthic community on the reef flat is less diverse than the reef slope, the reef flat habitat is important for trophodynamics of the entire reef system^26^.

Typically reef flats can be defined as either coral- or rubble-dominated, and within each reef flat clear zonation in the biological community can be observed from the high wave energy windward side of the reef to the low wave energy leeward side^24^. In the central Red Sea, the branching coral *Stylophora pistillata* is common in shallow areas and can be found across all reef flat zones^27–29^. *Stylophora pistillata* has been called an r-strategist because it quickly colonizes unstable environments, reproduces early in its lifetime, grows quickly, and has high population turnover^30^. These characteristics allow *S. pistillata* to be dominant in the disturbance-prone reef flat habitat, while in more stable environments it tends to be outcompeted by other coral species^30^. The reef flat habitat is therefore an ideal system to study *S. pistillata* size structure, as this coral’s high population turnover is likely to reflect recent natural or anthropogenic disturbance history.

Previous studies in the Red Sea have examined the abundance and size class structure of juvenile pocilliporid corals^31^ and common coral species over cross-shelf^32^ and large-scale gradients^28^. However, despite being the most abundant species on reef flats in the region^27^, no studies assessing size-class structure have been conducted on *S. pistillata* populations, and intra-reef comparisons are lacking. Such information is critical for understanding population dynamics in a region that is increasingly affected by coral bleaching events^33,34^. Here, we conduct a population structure assessment of *S. pistillata* by evaluating size structure, color morph distribution, and incidence of partial mortality to investigate large and small-scale patterns of variability across the shelf and within each reef across the reef flat.

## Materials and methods

### Study sites

**Figure 1:**
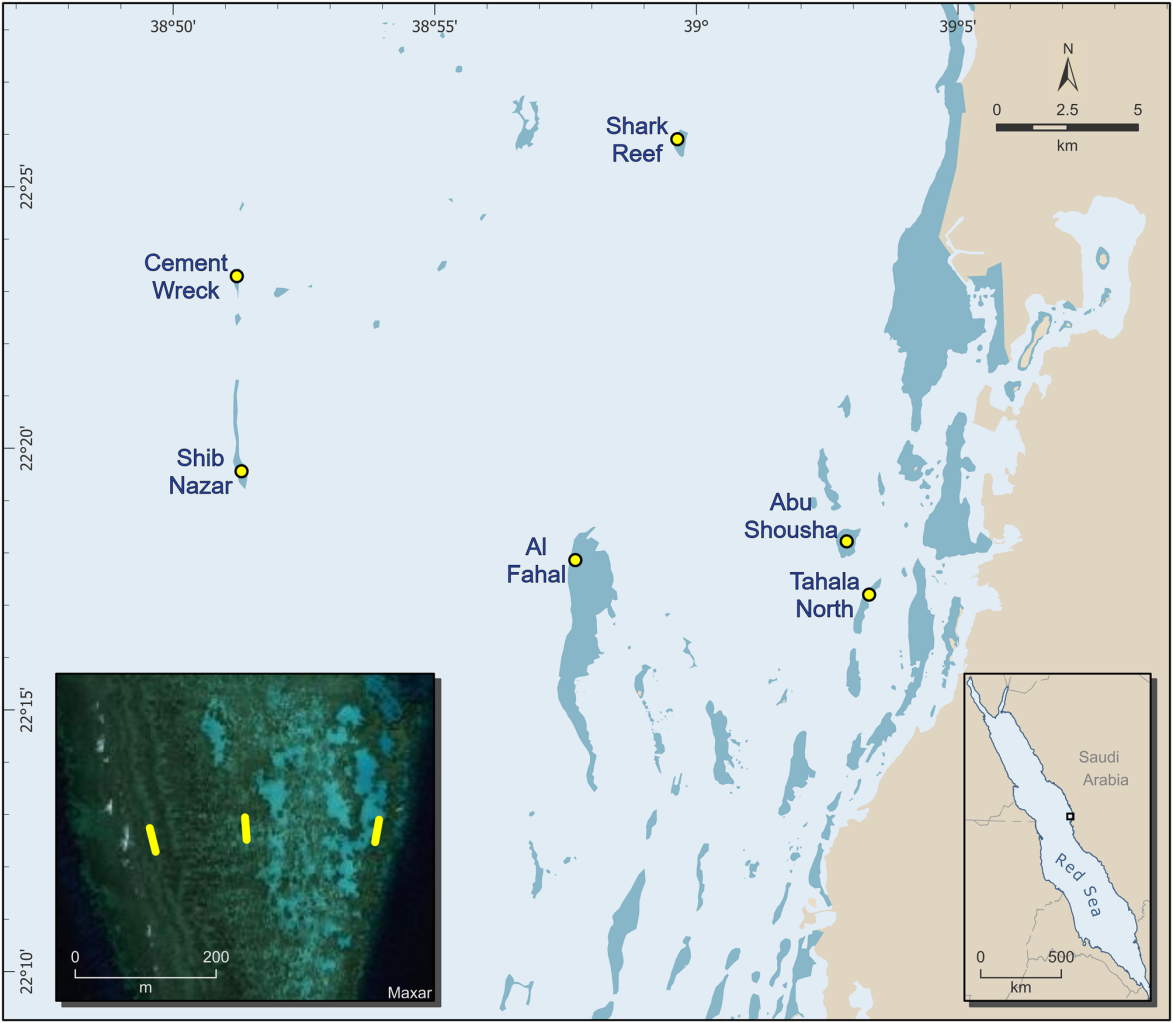
Map of the study area showing all reefs surveyed. Phototransects were conducted in the exposed, midreef and sheltered zones of the reef flat (left to right in the inset). The reef depicted in the inset is Shib Nazar.

**Figure 2:**
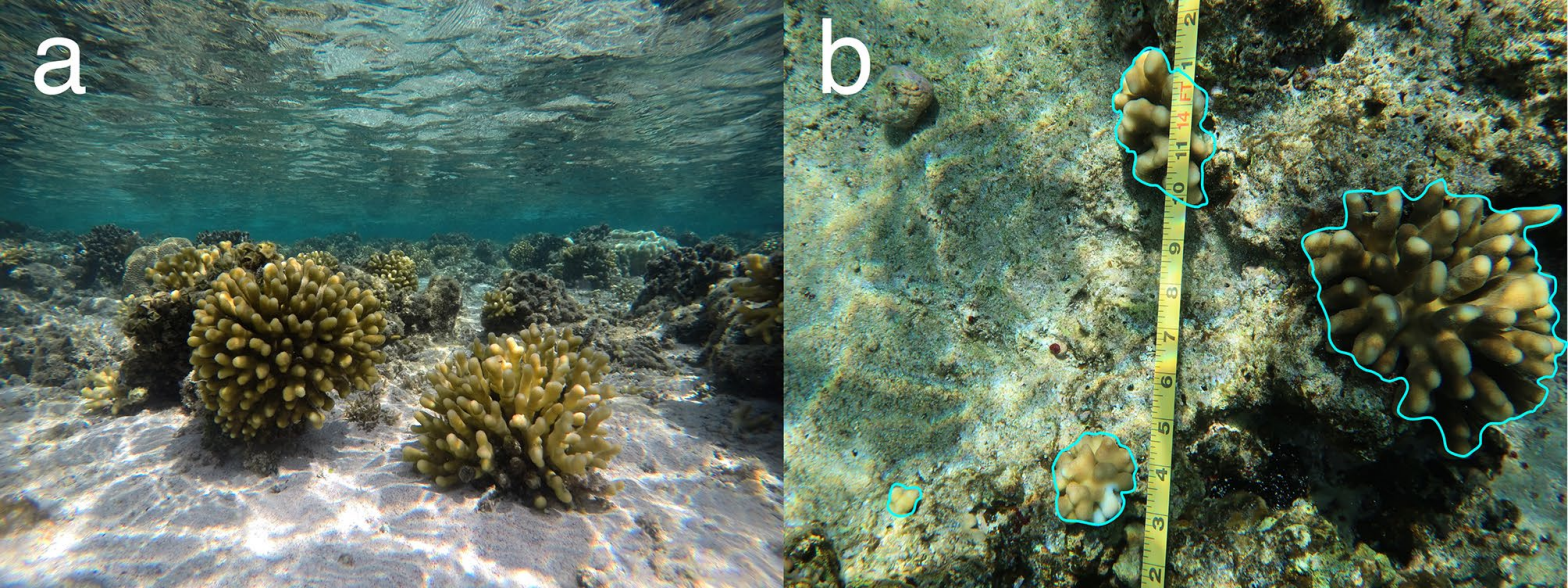
In situ photos of one of the reef flats surveyed in this study. (**a**) *Stylophora pistillata* is an abundant coral on central Red Sea reef flats. (**b**) An example of an image from the phototransects. Each coral in the image is measured with the tool ImageJ to obtain the planar area of the colony.

This study was carried out between 16–20 December 2020, on six reef flats in the central Red Sea over a cross-shelf gradient, with the closest reefs situated ~ 2 km from shore and the farthest ~ 20 km offshore (Fig. 1). We selected two nearshore (Abu Shousha and Tahala North), two midshelf (Al Fahal and Shark Reef), and two offshore (Cement Wreck and Shib Nazar) reefs for this study. The reef flats are characterized by consolidated pavement, rubble, or sand, and are dominated by the branching coral *S. pistillata* (Fig. 2a). The width of each reef flat (i.e., axis that is perpendicular to shore) varies from 125 to 300 m, and all are at depths between 50 and 150 cm. Within each reef, three sites were chosen to represent the exposed, midreef, and sheltered zones of the reef. The exposed sites receive the most wave action, and are typically on the western edge of the reef flat (within ~ 5 m in front of the breaking waves). The midreef sites are roughly halfway across the reef flat, while the sheltered sites are on the leeward reef edge, generally on the eastern side of the reef (Fig. 1 inset).

### Temperature profiles

As water flows across the reef flat, it warms during the day and can result in a temperature gradient from the exposed to sheltered sides^35^, which may influence the ecology of *S. pistillata* in different reef zones. As part of a separate ongoing study for monitoring fine-scale differences in temperature across reef flat zones, Onset Hobo pendant temperature loggers were deployed at each reef flat zone of all six reefs, starting in June 2019. The calibrated loggers were programed to log temperature every 10 min, and were secured to lead weights and deployed on the substrate at 50–100 cm depth. Both the weights and loggers were wrapped in white electrical tape to minimize the effects of intense sunlight in shallow water on the logger’s temperature readings. Loggers were swapped out every two to twelve months depending on logistical constraints (e.g., national COVID-19 lockdowns). Temperature was logged for some reefs starting in June 2019 and near-continuous profiles were obtained until the time of the study. Unfortunately, many sites are missing data for long periods of time due to logistical constraints. We therefore chose to focus on the two reefs with the most complete datasets: the offshore reef Shib Nazar and the nearshore reef Abu Shousha. We highlighted a six-week period of 2020 when temperatures were highest and calculated the degree-heating weeks for each reef zone. Degree-heating weeks is a measure of accumulated heat stress commonly used in coral biology^36^. We used a baseline of 31.8 °C, one degree above the mean maximum for this region, which has been used in previous studies for this area^37^.

### Phototransect surveys and image processing

At each of the reef sites, we conducted three replicate phototransect surveys of 10 m length. Transect locations were haphazardly selected to begin near the temperature logger deployed at each of the sites, and were laid parallel to the reef edge with 3–5 m separating the start and end of each replicate. Photos were taken along the entire length of the transect via snorkel using a Canon G7 X camera (Tokyo, Japan). Photos were later processed in the software ImageJ to count the number and size of *S. pistillata* colonies^38^. Size was measured by calibrating each image to a known length (referencing the transect tape in the photo) and using the “area” measurement tool in ImageJ to trace the contours of each colony, resulting in the planar area^39^ (Fig. 2b). All colonies of *S. pistillata* that fell completely within the area of the photo were counted and measured; colonies that were partially outside of the photo were not considered in the analysis. Many colonies suffered partial mortality; in these cases, a separate measurement was taken to quantify the amount of living tissue relative to the size of the colony. For measuring abundance, we only considered colonies with greater than 50% living tissue (sensu^19^). Since the water depth on reef flats varied slightly between reefs and with tidal oscillations, the distance between the camera from the substrate was not equal for all surveys, resulting in differences in the area covered for each photo. To standardize abundance counts between reefs, we measured the area of each photo to obtain the total area surveyed for each site, and expressed abundance in terms of density (individual colonies m^−2^). For all colonies considered in the analysis (> 50% living tissue), we also quantified partial mortality between sites and reefs. For each colony we also assigned a color morph category: yellow, purple, or mixed colors, if the colony was partially both colors. We also calculated the proportion of juvenile colonies based on previous studies that consider colonies of *S. pistillata* and related species less than 10 cm^2^^2^ planar area likely to be juveniles^11,40^. Finally, summing the total area of all colonies allowed us to calculate the percent cover of *S. pistillata* for each reef flat zone to compare with colony density.

### Data analysis

We plotted the size class structure of each reef site, the total for each reef, and the entire sampled population of *S. pistillata* in our study system. Colony size was first log transformed to normalize the distributions. We separated size classes into bins of 0.5 on the log scale for visualizing size structure (a dashed line was added at the juvenile adult cut-off, 10 cm^2^^41^). The descriptive statistics (skewness, kurtosis, mean size) were calculated for each site individually, as well as for each reef and the total population sampled; these analyses were performed in R (R Development Core Team 2018) using the package “moments”^42^. We compared log-transformed size-frequency distributions using two-sided Kolmogorov–Smirnov tests for reef zones within each reef. Due to extreme variability among reefs, we did not perform these tests for reef flat zones between different reefs. For each of the assessed parameters (e.g., density, mean size, etc.), we performed a non-parametric Kruskal–Wallis analysis to test for differences among reef flat zones, reefs, and shelf position. When significant differences were found, pairwise Mann–Whitney U tests were performed.

A Permutational Multivariate Analysis of Variance (PERMANOVA) was carried out in Primer v7 (Permanova + for Primer^43^). The model included three factors: shelf (fixed, orthogonal, three levels – nearshore, midshelf, offshore); reef (random, nested in shelf, two levels per shelf position); and reef flat zone (fixed, nested in reef, three levels – exposed, midreef, sheltered). The analysis was conducted on the distance matrix generated using the Euclidean distance and considering mean colony size, colony density, percentage of purple colonies, and percentage of colonies with partial mortality as variables. Percent juveniles were not included in the analysis as this parameter correlated strongly with mean colony size (r^2^ = 0.846, p < 0.05). Data was normalized and transformed (square root) before the analysis.

## Results

### Temperature profiles

**Figure 3:**
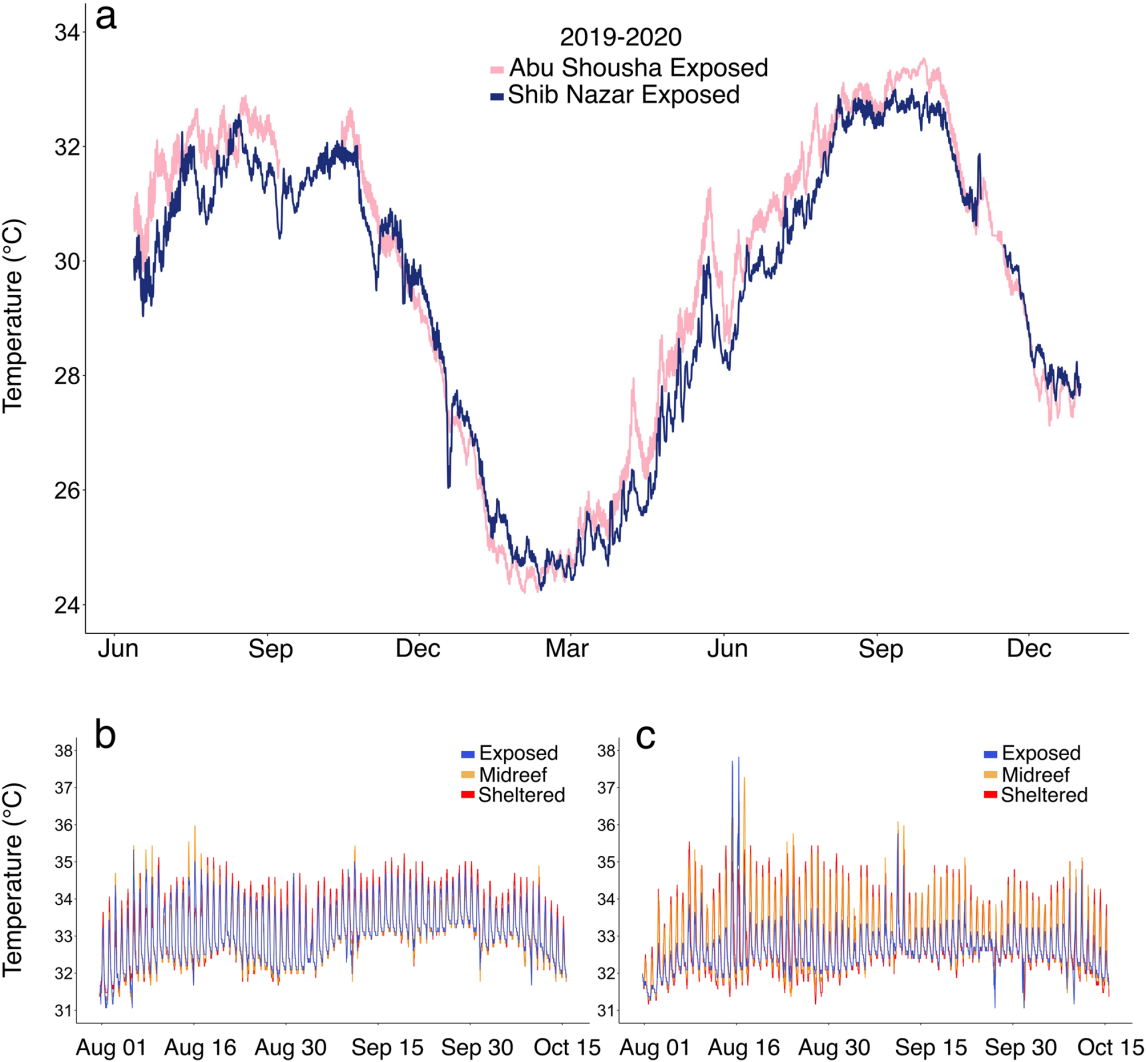
Temperature profiles of two of the reef flats in this study. (**a**) depicts an 18-month record of the exposed reef flat zones of the offshore reef Shib Nazar and the nearshore reef Abu Shousha (33-h low pass filter). The lower panels show diurnal temperature variation during the summer of 2020 at a 10-min frequency for Abu Shousha (**b**) and Shib Nazar (**c**) reef flats.

Overall, temperatures on the offshore reef Shib Nazar were lower than for the nearshore reef Abu Shousha. Comparing 18 months of temperature profiles of just the exposed reef flat zones of each reef shows that, generally, Abu Shousha experienced higher temperatures during peak summer months, but also lower temperatures during the winter months (Fig. 3a). To assess fine-scale variability across the reef flat zones, we focused on the warmest period of 2020, from August to mid October (Fig. 3b,c). Across reef zones within each reef, generally the exposed zone is less variable and has lower maximum temperatures than the midreef and sheltered reef flat zones, but this pattern is less pronounced for Abu Shousha (Fig. 3b) than for Shib Nazar (Fig. 3c). For this period, the mean daily temperature ranges of Abu Shousha are 1.88 ± 0.60°, 1.93 ± 0.80°, and 2.27 ± 0.44 °C (mean ± se) for the exposed, midreef and sheltered reef flat zones, respectively. For Shib Nazar, the values are 1.36 ± 0.96°, 2.46 ± 0.86°, and 2.68 ± 0.71 °C for the exposed, midreef and sheltered reef flat zones, respectively. Despite the differences in temperature variability, the mean temperatures during this period are similar across all reef flat zones of each reef (Abu Shousha: 33.10 ± 0.73°, 32.99 ± 0.73°, 33.11 ± 0.81 °C ; Shib Nazar: 32.61 ± 0.57°, 32.82 ± 0.87°, 32.88 ± 0.92 °C, exposed, midreef, and sheltered, respectively). The accumulated heat stress of Abu Shousha was higher than for Shib Nazar for all reef flat zones (Abu Shousha: 14.09, 12.92, 14.15 °C-weeks ; Shib Nazar: 8.66, 10.79, 11.58 °C-weeks, exposed, midreef, and sheltered, respectively).

### Coral size structure

Overall, an area of 279.16 m^2^ was surveyed in this study (Table 1). A total of 1,146 colonies of *S. pistillata* were counted across the study sites, ranging in size from 0.14 to 537.39 cm^2^ planar area (Table 1). The size structure is extremely variable, both across reef flat zones and between reefs, and there are no clear patterns across reef flat zones or reefs in terms of mean size. The highest mean colony size was 153.83 ± 34.32 cm^2^ at the exposed reef flat zone on Shark Reef, while the smallest mean colony size was found at the exposed reef flat zone of Al Fahal at just 6.37 ± 1.35 cm^2^ (Table 1), which are both midshelf reefs. Furthermore, within-reef mean colony sizes are variable and there is no clear trend between exposed, midreef or sheltered reef flat zones among reefs. For each individual reef, there were no differences in mean size across the different reef flat zones (Kruskal–Wallis, p > 0.05 for each). Reefs had significantly different mean colony sizes (Kruskal–Wallis, p < 0.05). Abu Shousha has the smallest mean colony size, Tahala North has the largest, and the other reefs had intermediate mean colony sizes. Considering shelf position, the midshelf reefs have significantly smaller mean colony sizes (39.99 ± 4.60 cm^2^) compared to the offshore reefs ( 60.27 ± 3.87 cm^2^) and nearshore reefs (50.28 ± 4.99 cm^2^) (Mann–Whitney U test, p < 0.05 for each). Offshore and nearshore reefs did not have significantly different colony sizes (Mann–Whitney U test, p = 0.583).

**Table 1:** Summary data of *Stylophora pistillata* populations from different reefs and reef flat zones (g1 is skewness; g2 is kurtosis).

Shelf position	Reef	Reef zone	Area surveyed (m^2^)	Density (ind. m^−2^)	% cover	Mean size ± SE (cm^2^)	Size range (cm^2^)	% juvenile colonies	% partial mortality	% purple colonies	g1	g2
Nearshore	Abu Shousha	Exposed	8.41	6.66	1.26	18.90 ± 3.64	0.26–142.78	57.14	7.14	8.93	− 0.13	2.62
		Midreef	7.37	10.44	1.94	18.58 ± 3.43	0.25–176.24	51.95	1.30	7.79	0.01	2.99
		Sheltered	9.26	8.31	4.29	51.63 ± 8.83	0.84–420.22	35.06	11.69	3.90	0.14	2.12
		Entire reef	25.05	8.38	2.58	30.78 ± 3.75	0.25–420.22	47.14	6.67	6.67	0.09	2.73
	Tahala North	Exposed	18.22	0.44	0.06	12.92 ± 5.82	4.47–52.98	75.00	0.00	25.00	1.46	4.05
		Midreef	16.89	3.26	4.11	126.36 ± 14.51	6.22–387.24	5.45	29.09	5.45	− 0.50	2.58
		Sheltered	13.98	1.29	1.73	134.04 ± 32.63	8.64–481.51	11.11	0.00	0.00	− 0.32	1.99
		Entire reef	49.08	1.65	1.93	116.86 ± 12.72	4.47–481.51	13.58	19.75	6.17	− 0.44	2.21
Midshelf	Al Fahal	Exposed	13.63	3.81	0.24	6.37 ± 1.35	0.59–55.73	86.54	3.85	80.77	0.56	2.90
		Midreef	20.46	2.20	0.35	16.10 ± 4.06	0.55–136.01	62.22	8.89	17.78	0.26	2.48
		Sheltered	17.41	6.61	3.59	54.33 ± 8.18	0.45–418.43	40.90	6.96	24.35	0.02	2.15
		Entire reef	51.49	4.12	1.42	34.45 ± 4.77	0.45–418.43	56.60	6.60	36.79	0.40	2.37
	Shark Reef	Exposed	17.24	0.87	1.34	153.83 ± 34.32	9.33–409.30	6.67	46.67	0.00	− 0.66	3.73
		Midreef	11.94	1.17	0.22	18.41 ± 4.42	2.13–59.74	50.00	14.29	0.00	− 0.01	2.35
		Sheltered	24.15	1.16	0.38	31.38 ± 8.17	1.02–212.13	46.43	7.14	0.00	0.08	1.78
		Entire reef	53.34	1.07	0.65	60.59 ± 12.26	1.02–409.30	36.84	19.30	0.00	0.02	2.06
Offshore	Cement Wreck	Exposed	22.84	4.03	4.19	104.08 ± 12.29	1.36–471.16	27.17	6.52	8.70	− 0.35	1.89
		Midreef	15.93	11.93	4.18	35.04 ± 4.44	0.14–351.31	46.84	11.58	0.53	− 0.13	2.95
		Sheltered	13.37	3.59	4.47	124.37 ± 18.43	2.11–437.98	18.75	33.33	0.00	− 0.50	2.06
		Entire reef	52.14	6.33	4.26	67.28 ± 5.41	0.14–471.16	37.27	13.33	2.73	− 0.14	2.42
	Shib Nazar	Exposed	16.25	8.43	3.39	40.20 ± 5.30	0.89–485.53	35.77	18.25	6.57	0.05	2.38
		Midreef	13.94	6.67	5.00	74.90 ± 12.20	0.4–537.39	40.86	13.98	0.00	− 0.06	2.14
		Sheltered	17.88	1.45	0.36	24.64 ± 6.67	0.7–124.76	57.69	3.85	19.23	0.21	1.67
		Entire reef	48.07	5.33	2.73	51.23 ± 5.41	0.4–537.39	39.84	15.23	5.47	0.02	2.33
Total population			279.16	4.11	2.49	54.11 ± 2.60	0.14–537.39	41.53	12.04	10.47	0.02	2.29

Density of *S. pistillata* was similarly variable over multiple spatial scales, and ranged from 0.44 colonies m^−2^ at the exposed reef flat zone of Tahala North to 11.93 colonies m^−2^ at the midreef reef flat zone of Cement Wreck (Table 1). For each individual reef, there were no differences in density across the different reef flat zones (Kruskal–Wallis, p > 0.05 for each). However, reefs did have statistically different densities, with Tahala North and Shark Reef having the lowest densities, Al Fahal, Cement Wreck and Shib Nazar having intermediate densities, and Abu Shousha having the highest density (Kruskal–Wallis, p < 0.05). The offshore and nearshore reefs have similar overall density of *S. pistillata* (5.85 and 3.93 individuals m^−2^, respectively; Mann–Whitney U test, p = 0.606), which is higher than the midshelf reefs (2.57 individuals m^−2^; Mann–Whitney U test, p < 0.05 for each).

**Figure 4:**
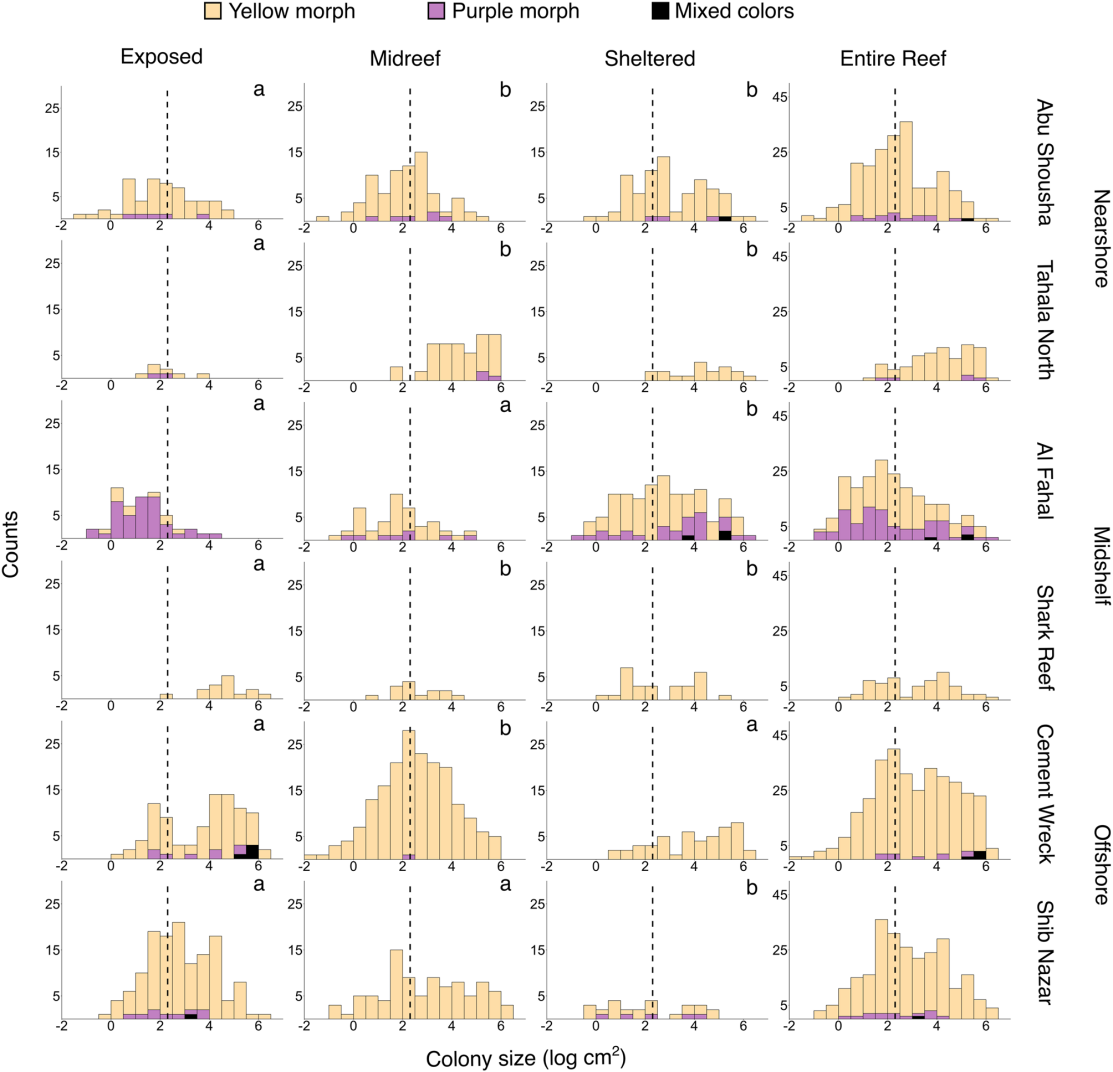
Size class structure of *Stylophora pistillata* colonies for each reef flat zone and each reef. The dashed vertical line represents the juvenile-adult cutoff of 10 cm^2^^41^. Bars are color-coded to show the proportion of colonies of different color morphs for each size class. Different letters between reef flat zones within each reef indicate significant differences in size-frequency distributions (two-sided Kolmogorov–Smirnov test). Distributions were not tested across different reefs.

**Figure 5:**
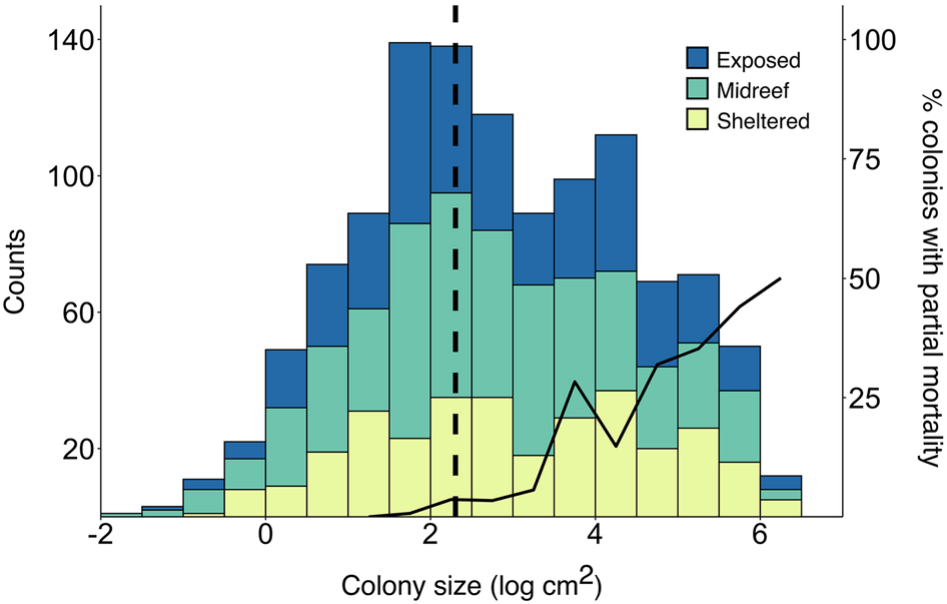
Size class structure of all *Stylophora pistillata* colonies counted in this study. The dashed vertical line represents the juvenile-adult cutoff of 10 cm^2^^41^. The solid line represents the percentage of colonies within a size class that suffered partial mortality. Bars are color coded to show the proportion of colonies found in different reef flat zones for each size class across all reefs surveyed in this study.

Considering size-frequency distributions for each site (Fig. 4) and the entire population sampled in this study (Fig. 5), we found that skewness (g_1_) ranged from − 0.66 at the Shark Reef exposed zone to 1.46 at the Tahala North exposed zone (Table 1). Abu Shousha showed a negatively skewed distribution for the exposed reef flat zone, but positively distributed for the midreef and exposed reef flat zones. Tahala North showed the opposite trend, with its exposed reef flat zone being the only negatively skewed zone. Al Fahal had positively skewed distributions for all reef flat zones, while the only positively skewed site for Shark Reef was the sheltered reef flat zone. Cement Wreck had negatively skewed distributions for all reef flat zones, while Shib Nazar only had a negative skew for the midreef reef flat zone. Considering the size frequency of entire reefs, two are negatively skewed (Tahala North and Shib Nazar) and the rest are positively skewed. Kurtosis (g_2_), which is a measure of the effect of outliers on a distribution, was variable among sites but mostly platykurtic (few outliers in the population; g_2_ < 3) or mesokurtic (roughly approximating a normal distribution; g_2_ ~ 3). The only cases where sites’ kurtosis was leptokurtic (many outliers in the population; g_2_ > 3) were the exposed reef flat zones of Tahala North and Shark Reef (Table 1). The Kolmogorov–Smirnov tests revealed that within every reef, there were differences in size-frequency distributions between one reef flat zone with the other two; however, there was no consistency of which reef flat zone was unique (exposed for Abu Shousha, Tahala North and Shark Reef; midreef for Cement Wreck; and sheltered for Al Fahal and Shib Nazar) (Fig. 4).

The proportion of juvenile colonies varied between reefs and reef flat zones. The highest percentage was observed at the exposed zone of Al Fahal, with 86.54% of colonies under 10 cm^2^ planar area, while the lowest was 5.45% at the midreef zone of Tahala North (p < 0.05, Mann–Whitney U test) (Table 1, Fig. 4). For each individual reef, there were no differences in proportion of juveniles across the different reef flat zones (Kruskal–Wallis, p > 0.05 for each). Reefs did differ in the proportion of juvenile colonies, with Tahala North and Al Fahal having higher proportions than the other reefs (Kruskal–Wallis, p < 0.05). The proportion of juveniles was not significantly different at the level of shelf position (Kruskal–Wallis, p = 0.198).

### Size structure compared to coral cover

**Figure 6:**
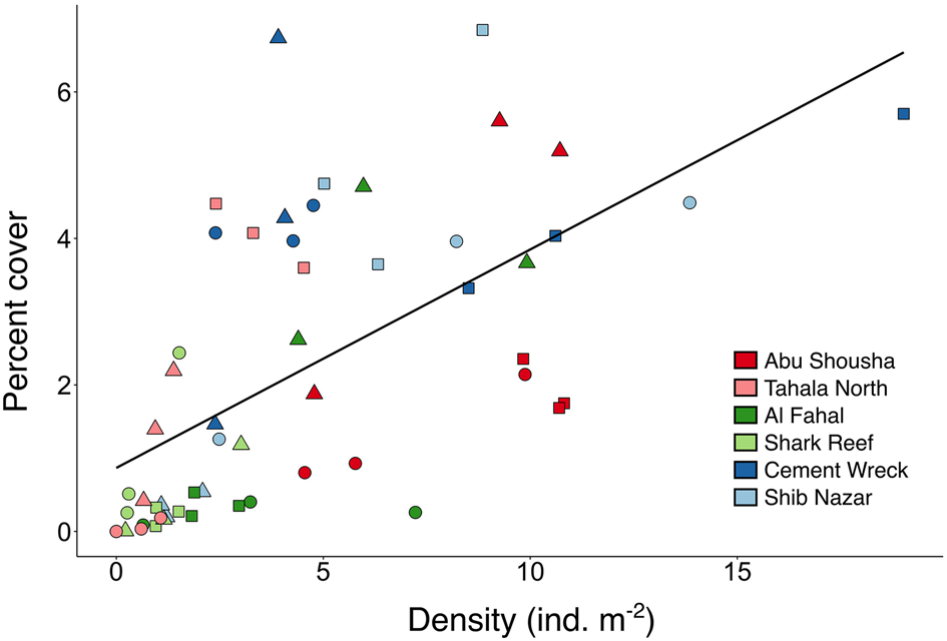
Density (ind. m^−2^) compared to percent cover of *S. pistillata* colonies across reef flat zones from six reefs (circles = exposed, squares = midreef, triangles = sheltered reef flat zones). Each point represents one replicate transect.

Summing the planar area of *S. pistillata* for each transect allows us to compare the percent cover to some aspects of size structure. Colony density is positively correlated with percent cover, but this correlation is weak (r^2^ = 0.373, p < 0.05) (Fig. 6). Since percent cover is a broad measurement that obscures more detailed information, sites with similar values of percent cover can have substantially different size class distributions. For example, the exposed and midreef reef flat zones of Cement Wreck have almost identical percent cover of *S. pistillata* (4.19 and 4.18%, respectively). However, the colony density of the exposed zone is nearly three times lower than the midreef zone (4.03 and 11.93 ind. m^−2^, respectively), and the mean colony sizes differ significantly (104.08 ± 12.29 and 35.04 ± 4.44 cm^2^, respectively; p < 0.05, Mann–Whitney U test). In addition, their size-frequency distributions are significantly different (Kolmogorov–Smirnov, p < 0.05).

### Frequency of colonies with partial mortality

Incidence of partial mortality ranged from 0 to 47% of colonies between reef flat zones (Table 1). There were no differences in frequency of partial mortality among reef flat zones, reefs, or shelf position (Kruskal–Wallis, p > 0.05 for each). Across the entire study there is a trend of increasing proportion of colonies with partial mortality with increasing size class (Fig. 5), reaching 50% for *S. pistillata* found in the largest size class.

### Color morph abundance and distribution

The proportion of purple colonies per site varied widely, from 0% at several sites to 81% in the exposed reef flat zone of Al Fahal. Mixed color morphs are uncommon, only accounting for 0.7% of colonies from three reefs. Overall, yellow colonies dominated reef flats in this study, accounting for nearly 90% of all colonies surveyed (Table 1). For each individual reef, there were no differences in proportion of purple colonies across the different reef flat zones (Kruskal–Wallis, p > 0.05 for each). However, there were differences at the level of reef (Kruskal–Wallis, p < 0.05), with Al Fahal having a higher of purple colonies than all other reefs (Mann Whitney U test, p < 0.05 for each). In additon, no purple colonies were found in Shark Reef, resulting in a lower proportion than all other reefs (Mann Whitney U test, p < 0.05 for each). Though not statistically significant (Kruskal–Wallis, p = 0.058), there is an overall trend of a higher percentage of purple colonies in the exposed zones (18.33%) than the other reef zones (11.54 and 3.80% for sheltered and midreef zones, respectively). There is no difference in the proportion of purple colonies at the level of shelf position (Kruskal–Wallis, p = 0.327).

### Population structure

As no broad trends were found in the analysis of coral size structure, a PERMANOVA analysis was conducted including data on partial mortality and color morph assignment. The PERMANOVA analysis showed no differences for the factor shelf, but a significant interaction between the factors reef and reef flat zone, showing a high level of variability within the sampling area. Pair-wise tests of reefs within each shelf position revealed that the offshore reefs Cement Wreck and Shib Nazar are not significantly different, but the nearshore (Abu Shousha and Tahala North) and midshelf (Al Fahal and Shark Reef) reefs are different from each other within groups. Considering reef flat zones within each reef, in Abu Shousha the reef flat zones were not significantly different from each other, while Tahala North, Al Fahal and Cement Wreck showed differences between all reef flat zones. Shark Reef only had significant differences between the exposed and sheltered reef flat zones, while Shib Nazar had significant differences between sheltered and both midreef and exposed reef flat zones (Table 2).

**Table 2:** Population structure

Source	Df	MS	Pseudo-F	P (perm)
Shelf	2	9.040	0.500	0.942
Reef (Shelf)	3	18.040	13.710	**0.001**
Reef zone (Reef(Shelf))	12	7.700	5.850	**0.001**
Pairwise for term Reef (Shelf)		t	P(MC)	
Within level (Nearshore)	Abu Shousha, Tahala North	4.152	**0.001**	
Within level (Midshelf)	Al Fahal, Shark Reef	4.399	**0.001**	
Within level (Offshore)	Cement Wreck, Shib Nazar	1.526	0.064	
Pairwise for term Reef x Reef zone (Reef(Shelf))		t	P(MC)	
Within level (Nearshore)				
Abu Shousha	Exposed, Midreef	1.252	0.265	
	Exposed, Sheltered	1.532	0.140	
	Midreef, Sheltered	2.158	0.056	
Tahala North	Exposed, Midreef	3.633	**0.004**	
	Exposed, Sheltered	3.097	**0.018**	
	Midreef, Sheltered	3.312	**0.005**	
Within level (Midshelf)				
Al Fahal	Exposed, Midreef	2.747	**0.015**	
	Exposed, Sheltered	2.513	**0.027**	
	Midreef, Sheltered	2.462	**0.030**	
Shark Reef	Exposed, Midreef	2.224	0.068	
	Exposed, Sheltered	3.065	**0.024**	
	Midreef, Sheltered	0.274	0.907	
Within level (Offshore)				
Cement Wreck	Exposed, Midreef	3.149	**0.027**	
	Exposed, Sheltered	2.213	**0.040**	
	Midreef, Sheltered	2.917	**0.013**	
Shib Nazar	Exposed, Midreef	1.215	0.279	
	Exposed, Sheltered	2.098	**0.036**	
	Midreef, Sheltered	2.927	**0.014**	

Results of the three-way PERMANOVA analysis based on the Euclidean distance and after normalization and transformation. The dataset across shelf, reef, and zone consisted of data from colony density, size, partial mortality, and color morphs. Significant effects at p < 0.05 are shown in bold.

## Discussion

Assessments of size class structure can yield insights into coral population status^5,6^. Using size frequency distributions instead of percent coral cover is more informative for understanding demographic trajectories. In this study, we found large variability of the size class structure of *S. pistillata* both within and among reefs. We did not observe a clear pattern for either cross-shelf or within-reef scales for any of the parameters analyzed separately (colony densities, size, partial mortality, and color morphs). However, multivariate analysis conducted on these parameters showed that most reefs had significant differences between some or all of their reef flat zones. Our results for two reefs with differing thermal profiles suggest that environmental factors such as temperature may be partly responsible for driving spatial differences in *S. pistillata* population structure. In the Red Sea, few historical datasets are available, especially outside of the northern Gulf of Aqaba region. Although *S. pistillata* from this region tend to be resilient to high temperatures, further warming due to climate change may result in temperatures that exceed their thermal tolerance, especially when coupled with local stressors like pollutants^44^. This demographic “snapshot” will therefore serve as a baseline assessment of this common species, which is essential for future biomonitoring. Such baselines have revealed changes in size structure of *Stylophora* corals in other regions. For example, in the Great Barrier Reef, the genus *Stylophora* has experienced major declines in overall abundance over the past two decades, with concomitant shifts in size class distribution^10^. In Kenya, *Stylophora* underwent a significant reduction in median colony size following the mass bleaching event of 1998^12^.

### Temperature variability in space and time

The within-reef patterns of temperature were broadly similar between the two reefs assessed. The midreef and sheltered reef flat zones tended to have larger daily ranges and higher maximum temperatures than the exposed reef flat zones of both reefs (Fig. 3b,c). In addition, for both reefs, the accumulated heat stress was highest at the sheltered reef flat zone. However, the offshore reef Shib Nazar exhibited a more pronounced difference between the exposed and other reef flat zones, which is reflected in the accumulated heat stress (the midreef and sheltered reef flat zones are 25 and 34% higher, respectively, than the exposed reef flat zone) (Fig. 3c). Interestingly, for Abu Shousha, the midreef reef flat zone had the lowest accumulated heat stress (9% lower than the exposed reef flat zone), while the sheltered reef flat zone was similar to the exposed reef flat zone (0.5% higher). The difference in variability between the two reefs may be due to two phenomena: the shelf position of each reef, and the distance across each reef flat (Shib Nazar, ~ 300 m; Abu Shousha, ~ 140 m). As an offshore reef, Shib Nazar receives wave-driven water flow from deeper, more thermally stable waters, particularly on the exposed side. Abu Shousha, on the other hand, is surrounded by shallow coastal waters that are subjected to greater temperature variability, even on the exposed side of the reef. This is supported by comparing the long-term temperature profiles of each reef’s exposed reef flat zone, where Shib Nazar tends to have less extreme temperatures in both summer and winter (Fig. 3a). In addition, the greater distance across Shib Nazar results in a longer residence time of seawater, allowing for greater diurnal heating potential, while the reef flat of Abu Shousha may not be large enough to induce such variation (i.e., the residence time is too short to cause substantial heating). This may explain why the midreef and sheltered reef flat zones of Shib Nazar tend to have much higher variability compared to the exposed reef flat zone, while all reef flat zones of Abu Shousha exhibit similar variability (Fig. 3b,c).

### Size structure

The results presented here offer a snapshot of *S. pistillata* populations on several reef flats in the central Red Sea. The information contained in the size frequency distributions reveal extreme variability on both inter-and intra-reef scales. We chose to use belt transects as a survey method, which may have a small bias towards counting smaller colonies. However, based on our personal observations, we are confident our results accurately reflect the true size structure. Previous studies of coral populations have also found variable size class structure across sites. Adjeroud et al. (2007) note that although they found significant variation in the size structure of two coral species in Moorea, they found no clear patterns in depth or location that could explain the differences, and suggest that other stochastic processes may be at play.

There are few patterns in any of the measured parameters at the level of reef flat zones, and over the entire population there is a fairly even distribution of all size classes among reef flat zones (Fig. 5). The high overall variability may be indicative of stochastic processes occurring in the area, as has been suggested in previous studies^19,20^. Considering shelf position, the offshore reefs have the highest density and largest colonies of *S. pistillata,* perhaps indicating these are more suitable habitats for sustaining larger populations of *S. pistillata* than the reefs closer to shore (Table 1). A paucity of smaller colonies suggests either a lack of recruitment or high mortality of younger colonies. Unlike many coral species which spawn, *S. pistillata* is a brooding coral that retains its larvae in the parent colony until planulation, and the majority of planula tend to settle within hours^45,46^. This suggests *S. pistillata* may have limited dispersal ability compared to broadcast spawning coral species. Indeed, some studies conducted in the same area as this study have suggested that there is genetic structure of *S. pistillata* among reefs^47^. The percentage of juveniles in different reef flat zones seems to change over the cross-shelf gradient, from more juveniles on the exposed reef flat zones of Abu Shousha, Tahala North and Al Fahal, to a lower proportion of juveniles on the exposed reef flat zones of Shark Reef, Cement Wreck and Shib Nazar (Table 1). It is unclear what causes this pattern, or if it simply is a stochastic process. Though not directly measured here, in our experience visiting these sites, the exposed reef flat zones of Shark Reef, Cement Wreck and Shib Nazar tend to experience higher wave action, potentially affecting settlement rates of *S. pistillata* on these reefs^48^.

Evaluating the shape (i.e., skewness) of the size class distribution can reveal important details about population structure. Considering the sites individually, half are negatively skewed, indicating a lack of smaller colonies and over-abundance of large colonies (Table 1). However, considering the reef as one entity, only two of six are negatively skewed, and the entire population is slightly positively skewed, showing that the scale of the survey is important for interpreting trends. Negatively skewed populations, with a lower proportion of small individuals, have been suggested to be indicative of a degraded environment^14^. However, some studies suggest that coral populations in degraded environments can also be positively skewed due to high adult mortality^12,28,49^. Without repeated monitoring, it is difficult to determine the drivers of skewness observed here.

Two of the reefs in this study are of note and deserve further discussion. Tahala North exhibits different substrate characteristics than the other reefs, with a layer of sand covering the pavement across most of the reef flat. Although *S. pistillata* is still common across much of the reef, the sandy substrate may influence growth and juvenile mortality, which affects population density. In addition, the exposed zone of Tahala North experiences seasonal *Sargassum* blooms which can completely cover any corals growing there for several months per year. We speculate the extremely low density of *S. pistillata* colonies there is due to periodic *Sargassum* blooms which may negatively affect *S. pistillata* growth^50^ or recruitment^51^. The second reef which deserves more attention is Shark Reef, which also had low overall density of *S. pistillata*. During the late summer of 2020, a bleaching event affected the reef system in the study region, but Shark Reef was the most impacted reef (pers. obvs.). During an October 2020 research expedition, we noted parts of the reef crest on Shark Reef experienced 100% bleaching (Fig. S2), whereas the other reefs in this study were minimally impacted. While we were not able to assess the reef flat on this expedition, it’s possible that habitat was also heavily impacted and the population of *S. pistillata* could have experienced high mortality rates. Interestingly, during a mass bleaching event in the central Red Sea in 2015/2016, Shark Reef (refered to as Qita Al-Kirsh) was also heavily impacted by bleaching, moreso than other midshelf and offshore reefs assessed^34^. This may be an indication that Shark Reef is more susceptible to disturbance, although it is not clear what factors may be responsible.

### Frequency of partial mortality

As modular organisms, corals frequently suffer partial mortality^52^, and the percentage of colonies with partial mortality tends to increase with increasing colony size^14,53–55^. Our results support this pattern, with the smallest size classes containing no colonies suffering partial mortality and an increasing proportion of partial mortality with increasing size class (Fig. 5). Furthermore, the site with the highest percentage of colonies with partial mortality is the exposed reef flat zone of Shark Reef, which also exhibits the largest mean size of any site in the study (Table 1). The largest size class in this study (*S. pistillata* larger than ~ 400 cm^2^) reached the highest incidence of partial mortality of 50% (Fig. 5), which is comparable to previous studies on other coral species^14^. Partial mortality has been shown to have little effect on per-polyp reproductive output^56^, meaning overall reproduction will decline proportional to tissue loss. As protoanderous simultaneous hermaphrodites, *S. pistillata* tends to produce only male gametes at smaller sizes (diameter < 4 cm) and produce both male and female gametes at larger sizes (diameter > 4 cm)^41^. Approximating the colony’s planar area as a circle, this equates to a cutoff of roughly 12.57 cm^2^, meaning only the smallest adults will be producing male-only gametes. Given that our results show the frequency of partial mortality is very rare in colonies of small size classes, partial mortality is unlikely to drastically affect the ratio of male/female gametes produced by *S. pistillata* colonies.

### Color morphs

Purple colonies accounted for just 10% of *S. pistillata* colonies over the entire study are. The dominance of yellow colonies on these reef flats is notable, as previous work has shown that purple morphs of *S. pistillata* typically outcompete the yellow morphs^57^. However, a related species that also exhibits yellow and purple color morphs (*Pocillipora damnicornis*) shows a similar pattern of yellow morphs dominating shallow areas and purple colonies more common below 3 m depth^58^. The spatial distribution of color morphs of other coral species are often related to the light environment^59^, and can influence disease or bleaching susceptibility^60,61^. However, the biological significance of color morphs in *S. pistillata* is not clear. Studies on the photochemistry of the pocilliporin pigments, which give the purple morphs their characteristic color, showed no evidence of photoprotection, function as an accessory pigment, or UV protection^62^. It may be that the yellow morphs of *S. pistillata* are better adapted to warmer temperatures, higher irradiances, or some other factor unique to reef flat environments, and the purple morphs are competitively dominant only in deeper areas. Color morphs may also harbor different symbiont communities, which may have different tolerances to temperature and irradiance^59^. Although *S. pistillata* from the Red Sea tend to exhibit a depth zonation in their Symbiodiniaceae communities (*Symbiodinium* in shallow habitats and *Cladacopium* in deeper habitats), a recent study conducted in the same area as our study found that both genera can be present in shallow-water colonies^63^. Regardless, the high percentage of purple morphs on Al Fahal is notable. The exposed reef flat zone is dominated by juvenile purple *S. pistillata*, which could indicate a recent recruitment pulse for that site. However, there is also a high proportion of adult purple colonies across all reef flat zones of Al Fahal. The higher abundance of purple morphs here may be the result of other stochastic processes.

### Multivariate analysis

While each of the individual factors examined here do not show any clear spatial patterns, the PERMANOVA analysis reveals most reefs have significantly different population structures across their reef flat zones. The different pattern of within-reef temperature variability of Abu Shosuha and Shib Nazar may help explain some patterns observed in the populations of *S. pistillata* at these sites. The multivariate analysis revealed that Abu Shousha was the only reef assessed which did not have significant differences between its reef flat zones. While other factors are likely also responsible, the similarity in temperature profiles between Abu Shousha’s reef flat zones may indicate that the environmental conditions are similar across the entire reef flat, which is reflected in the similarity of populations of *S. pistillata*. On the other hand, the sheltered reef flat zone of Shib Nazar was different from both the midreef and exposed reef flat zones (Table 2). Unfortunately we lack contemporaneous temperature data for the other reefs in this study, making it difficult to ascertain the importance of temperature on population structure at each site. However, these two contrasting patterns in terms of temperature variability within a reef flat indicates that environmental conditions can vary at small spatial scales, and may partly explain the lack of biological patterns observed in the asssessment of the population structure of *S. pistillata* in this study.

## Conclusion

As the dominant branching coral species inhabitating reef flats in the region, *S. pistillata* plays an important role in the ecology and geology of this sometimes overlooked habitat. Here we show that there is high variability over small spatial scales of size structure of this species, perhaps in large part driven by stochastic processes. It is also important to highlight that a multivariate analysis can reveal differences in populations which are not apparent in analyzing each parameter seperately. Our comparison of percent cover to colony density shows that the former metric obscures detailed information about the state of a population that can be gleaned from size structure data. Though our surveys reveal the offshore reefs may have “healthier” (higher colony density and mean colony size) populations of *S. pistillata*, future monitoring is needed to track population changes and relate them to potential disturbances (e.g., bleaching events, pollution, etc.). We hope the data presented here will foster future studies addressing which other abiotic processes and variables may play a role in this small-scale variability of size structure. Such information may help explain why some reefs experience mass bleaching of corals while other nearby reefs are largely spared. Finally, evaluating the role of biotic interactions (e.g., symbiodiniaceae community, microbiome, predators/herbivores, competition for space, etc.) at different spatial scales may also shed light on differences in population structure of *S. pistillata* in the region.

## Supplementary Information

Supplementary Information.

## Data Availability

The datasets used during the current study are available from the corresponding author on reasonable request.
