# Supplementary material for: Size structure of the coral Stylophora pistillata across reef flat zones in the central Red Sea
Source: Sci Rep. 2022 Aug 17;12:13979. doi: 10.1038/s41598-022-17908-3 (PMC9383669; doi:10.1038/s41598-022-17908-3)
Supplement: Supplementary file 1 — Supplementary Information. [file 41598_2022_17908_MOESM1_ESM.docx]

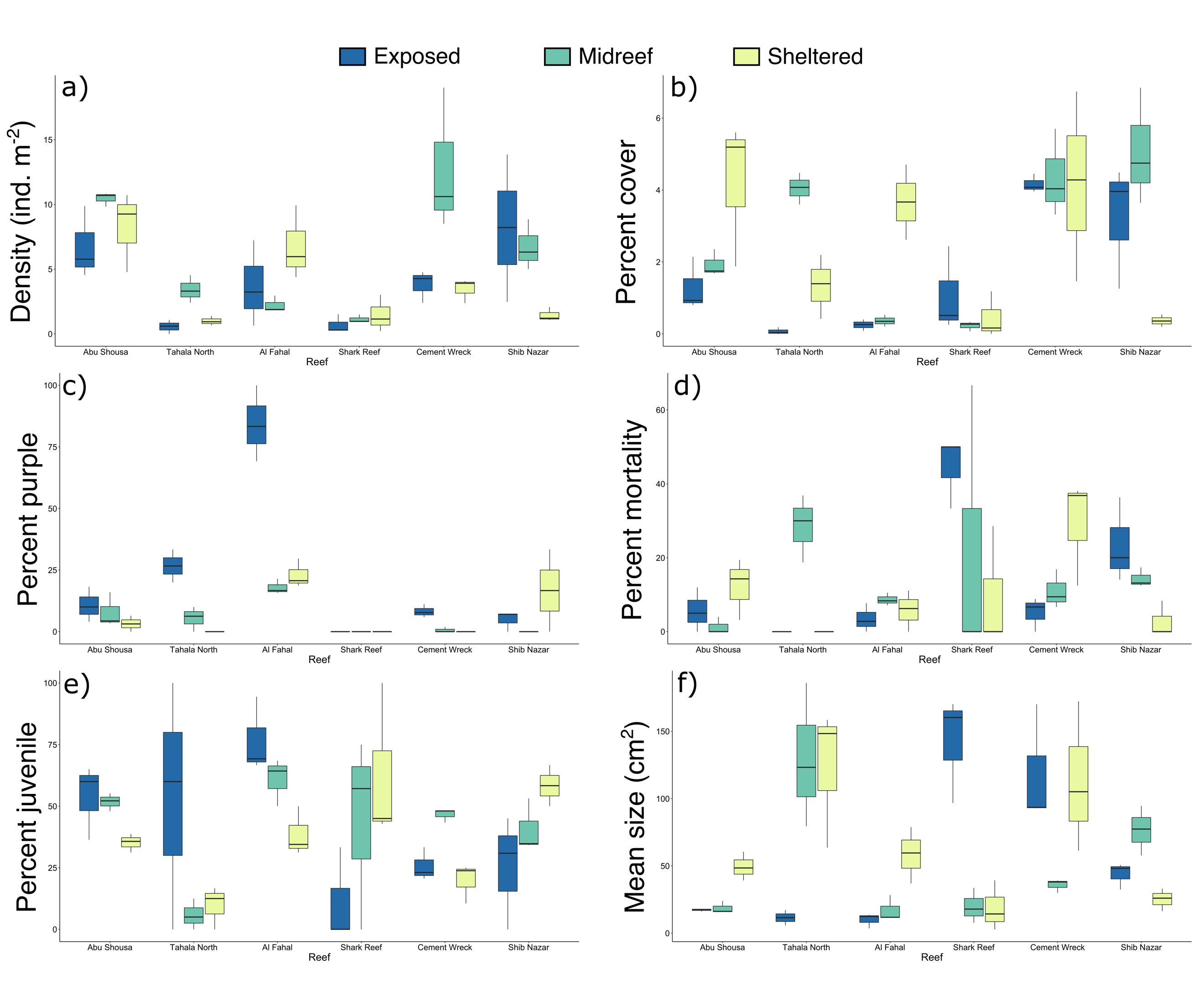


S1: All investigated parameters for each reef and reef flat zone, including colony density (a), percent cover (b), proportion of purple colonies (c), proportion of colonies suffering partial mortality (d), proportion of juvenile colonies (e), and mean size (f). Boxplots comprise three replicate transects.


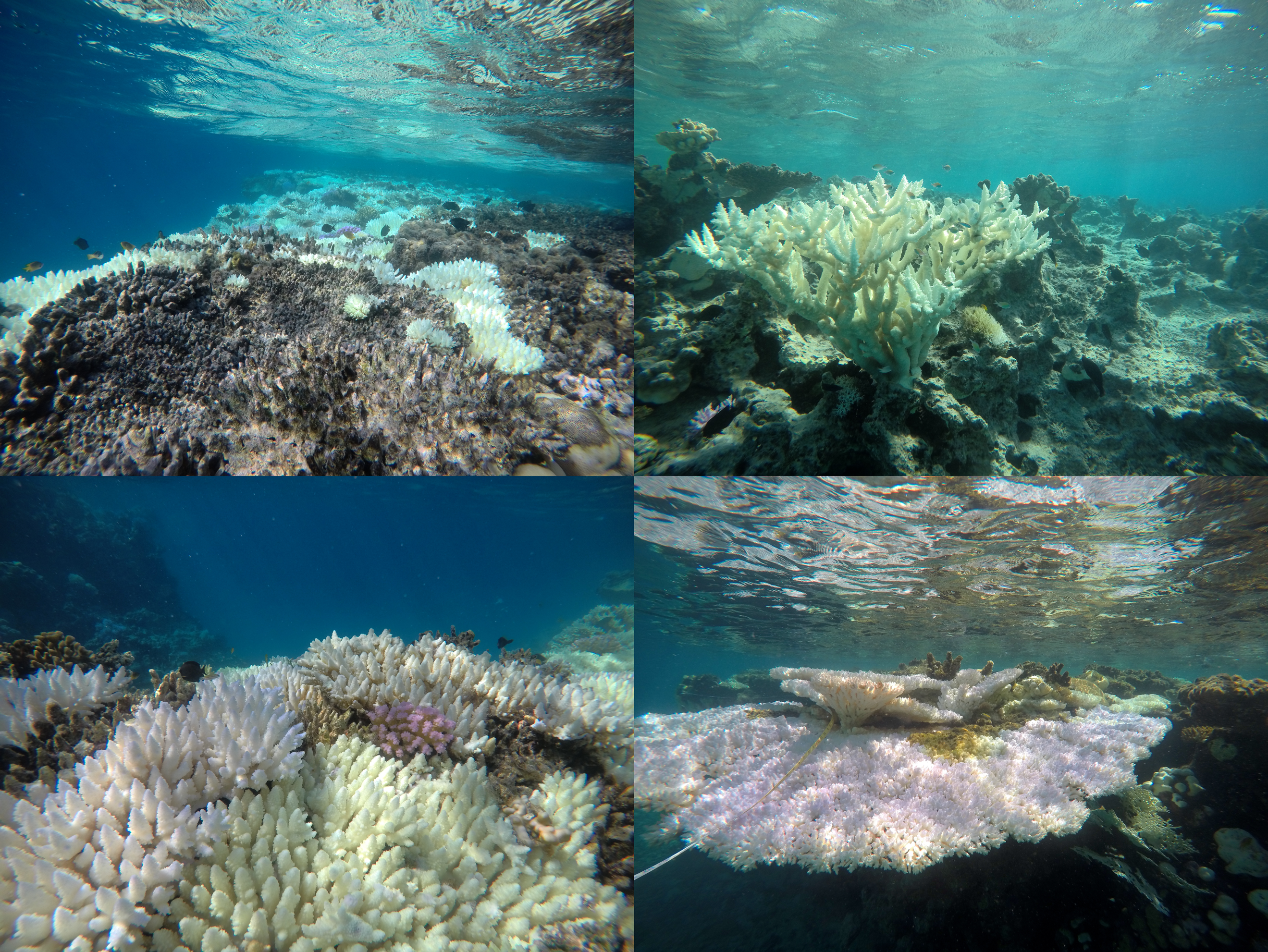


S2: *In situ* observations of a bleaching event on the fore reef of Shark Reef (outside of the study area surveyed in this study). The images were captured on October 15-16, 2021.
